# Hazard identification and characterization of leachable chemicals from plastic products – a new PARC project

**DOI:** 10.3389/ftox.2025.1719035

**Published:** 2025-11-27

**Authors:** Hubert Dirven, Aleksandra Bogusz, Hans Bouwmeester, Mathias Busch, Guillaume Duflos, Gunnar S. Eriksen, Margarida Fardilha, Daniela Flores-Gomez, Nina Franko, Laurent Gaté, Yves Guichard, Maria João Silva, Jorke H. Kamstra, Konstantinos M. Kasiotis, Sunmi Kim, Young Jun Kim, Youngsam Kim, Elise van der Koogh, Susana Loureiro, Henriqueta Louro, Kyriaki Machera, Raymond H. H. Pieters, Anastasia Spyropoulou, Evangelia N. Tzanetou, Catarina Malheiro, Tim Ravnjak, Guillermo Repetto, Gilles Rivière, Chang Seon Ryu, Evgenia Anna Papadopoulou, Konstantinos A. Aliferis, Anita Solhaug, Marija Sollner Dolenc, Martina Štampar, Ana M. Tavares, Knut Erik Tollefsen, Célia Ventura, Radoslaw Walkowiak, Walter Zobl, Bojana Žegura, Igor Snapkow, Dorte Herzke

**Affiliations:** 1 Department of Chemical Toxicology and Department of Food Safety, Norwegian Institute of Public Health, Oslo, Norway; 2 Institute of Environmental Protection, National Research Institute, Warszawa, Poland; 3 Division of Toxicology Wageningen University, Wageningen University and Research, Wageningen, Netherlands; 4 French Agency for Food, Environmental and Occupational Health & Safety (ANSES), Fougères, France; 5 Norwegian Veterinary Institute (NVI), Oslo, Norway; 6 Department of Medical Sciences, Institute of Biomedicine, University of Aveiro, Aveiro, Portugal; 7 Department of Population Health Sciences, Faculty of Veterinary Medicine, Institute for Risk Assessment Sciences, Utrecht University, Utrecht, Netherlands; 8 Faculty of Pharmacy, University of Ljubljana, Ljubljana, Slovenia; 9 Département de Toxicologie et Biométrologie, Institut National de Recherche et de Sécurité, Vandoeuvre-lès-Nancy, France; 10 National Institute of Health Dr. Ricardo Jorge, Departments of Human Genetics and Comprehensive Health Research Centre (CHRC), NOVA Medical School, NOVA University of Lisbon, Lisbon, Portugal; 11 Laboratory of Pesticides’ Toxicology, Scientific Directorate of Pesticides Control and Phytopharmacy, Benaki Phytopathological Institute, Benaki, Greece; 12 Chemical Analysis Center, Chemical Platform Technology Division, Korea Research Institute of Chemical Technology, Daejeon, Republic of Korea; 13 Korean Institute of Science and Technology Europe, Saarbrücken, Germany; 14 CESAM - Centre of Environmental and Marine Studies & Department of Biology, University of Aveiro, Aveiro, Portugal; 15 Department of Genetic Toxicology and Cancer Biology, National Institute of Biology, Ljubljana, Slovenia; 16 Universidad Pablo de Olavide (UPO), Seville, Spain; 17 Laboratory of Pesticide Science, Department of Crop Science, Agricultural University of Athens, Athens, Greece; 18 Department of Plant Science, Macdonald Campus of McGill University, Montreal, QC, Canada; 19 Norwegian Institute for Water Research (NIVA), Oslo, Norway

**Keywords:** plastics, chemicals, leachables, PARC, new approach methodologies, hazard assessment, toxicity, risk assessment

## Abstract

A recent study has suggested that plastics may contain more than 16,000 chemicals, including additives, processing aids, starting substances, intermediates and Non-Intentionally Added Substances. Plastic chemicals are released throughout the plastic life cycle, from production, use, disposal and recycling. Most of these chemicals have not been studied for potential hazardous properties for humans and in the environment. To refine the risk assessment of these leachable chemicals, additional hazard data are needed. The PlasticLeach project within the EU co-funded Partnership for the Assessment of Risks from Chemicals (PARC) aims to address this data gap by screening several plastic products in daily use. Leachates will be prepared from a number of these plastic items, and these chemical mixtures will be further tested using several test guideline compliant assays and New Approach Methodologies covering both human health and environmental endpoints. The most toxic leachates will be characterized using a non-targeted analysis pipeline to identify chemicals in the leachate. When single chemicals of concern are identified, these will be further tested to determine hazardous properties and identify the respective potency factors to better understand their specific hazard profiles. A tiered approach for hazard testing will be followed. The experimental work will be complemented by *in silico* toxicological profiling, using publicly available toxicity databases and tools, including Artificial Intelligence tools that cover both human and environmental endpoints. A comprehensive array of endpoints, including cytotoxicity, endocrine disruption, genotoxicity, immunotoxicity, reproductive toxicity and effects related to ecotoxicity will be evaluated. In this paper, we outline the plastic products to be tested and the battery of assays that will be used to identify hazards relevant to both human health and the environment. Data generated from *in silico*, *in vitro*, and *in vivo* approaches will be reported using standardized formats, stored within a centralized repository, and harmonized to adhere to the FAIR data principles (Findable, Accessible, Interoperable, and Reusable). This integrated strategy will not only advance our understanding of the risks associated with plastic-derived chemicals but will also provide critical support for regulatory decision-making and facilitate the development of safer, and more ecofriendly plastic materials in the future.

## 1 Introduction

We are living in the plastic age, and plastic materials have become indispensable in modern society due to their versatility, resistance to degradation, and low production costs. Plastics are widely used in products such as food packaging materials, synthetic textiles, personal care products, medical devices, drinking water pipes, toys and electronics, making them an integral part of daily life in western society. Global plastic consumption is substantial and cumulative production of primary (or virgin) plastic has grown from 2 megatonnes (Mt) in 1950 to 475 Mt in 2022. Without intervention, it is projected that global plastic production will nearly triple in 2060. The majority of all plastics produced is either still in use, discarded in landfills or mismanaged in the environment (Landrigan et al., 2025; Landrigan et al., 2023; Monclús et al., 2025; Weber et al., 2023). On a global scale less than 10% of plastics is recycled. These low numbers are due to recycling-related costs and due to fears that hazardous chemicals in plastics might be also recycled.

Plastics are manufactured from different polymer types, with Polyethylene terephthalate (PET), High-density polyethylene (HDPE), Low-density polyethylene (LDPE), Polyvinyl chloride (PVC), Polypropylene (PP), Polystyrene (PS), Polycarbonate and rubber (natural and/or synthetic or mixtures) being used in high volumes. While polymers themselves are generally considered to pose low concern due to their high molecular weight and are therefore exempted from registration and evaluation under REACH regulations, this assumption does not account for other substances present in plastic products. A wide range of chemical additives are incorporated into plastics to enhance their performance and durability, including plasticizers, fillers, flame retardants, antioxidants, heat stabilizers, colorants, lubricants and others (Weber et al., 2023).

Most plastics are produced from fossil-based resources, but recently biobased and/or biodegradable alternatives have been introduced. Biobased plastics usually refer to plastics from renewable biological resources, while biodegradable plastics are plastics that degrade by the actions of natural microorganisms such as algae, bacteria and fungi. (Nizamuddin et al., 2024). Example of bio-based plastics are polysaccharides, including cellulose and starch derivates. Polylactic acid (PLA) and starch are examples of bio-based and biodegradable plastics.

Plastics are not inert materials. Over time, they can be degraded and fragmented into micro- and nanoplastics (MNPs) and can leach chemicals during their degradation or aging process. Micro- and nanoplastics have a widespread distribution in the environment, and a recent study estimated that 27 million tons of nanoplastics are present in the Atlantic Ocean, suggesting an extremely massive, persistent and highly mobile contamination (Ten Hietbrink et al., 2025). Adverse health effects of micro- and nanoplastics in humans are considered likely but a knowledge-based risk assessment cannot be conducted yet due to knowledge gaps (Lamoree et al., 2025).

In addition to intentionally added substances, plastics may also contain non-intentionally added substances, such as degradation products of polymers and additives, impurities, reaction by-products and contaminants formed or introduced during recycling processes (Monclús et al., 2025). It has been hypothesised that the ageing or weathering of plastics materials may be a contributing factor to elevated levels of leachates (Luo et al., 2022).

Human and environmental monitoring studies across Europe have demonstrated that plastic materials can release complex mixtures of chemicals, resulting in widespread environmental and human exposure to phthalates, bisphenols, per- and polyfluoroalkyl substances (PFASs), chemicals that most likely are released from plastics (HBM4EU dashboard and the Norman database) (European Human Biomonitoring Dashboard, 2025; Norman, 2025; Paulsen et al., 2025).

As global concern over plastic pollution intensified, numerous initiatives have been launched to investigate the potential hazardous effects associated with plastics, especially considering the ongoing discussions on a United Nations treaty on plastic pollution as a result of UNEA resolution 5/14. This treaty is intended to address the entire life cycle of plastics, from production to disposal.

The European Chemicals Agency (ECHA) has released the PlasI database (ECHA, 2025), which provides information on substance names and CAS numbers, additive functions, and polymer types, covering 416 additives. However, the PlastChem Report outlines that plastics contain far more chemicals than previously recognized, identifying 16,325 chemicals included in the new PlastChem database that accompanies the report (Monclús et al., 2025). Six percent of these chemicals are internationally regulated under the Basel, Stockholm, and Minamata Conventions, and the Montreal Protocol, while 4,200 chemicals (26%) are considered of concern due to their persistent, bioaccumulative and toxic (PBT) properties, posing potential hazards to both human health and the environment. The report highlights 15 priority groups of concern, including aromatic amines, aralkyl aldehydes and alkylphenols, bisphenols, phthalates, chlorinated paraffins and per- and poly fluoroalkyls (PFAS). Most of these chemicals are not covalently bound to the polymer matrix and can migrate out of plastics when in contact with media like food, body fluids, as well as other materials and environmental compartments. Consequently, likely human exposure includes the oral, inhalation, and dermal routes. Likewise, wildlife exposure is anticipated to be through similar exposure routes, albeit exposure pathways may diverge considerably, based on what pollution sources are the most relevant and inherent physico-chemical properties governing the environmental fate and behavior (EPA, 2016) This report can be found at https://www.epa.gov/trash-free-waters/state-science-white-paper-effects-plastics-pollution-aquatic-life-and-aquatic.

Norner, an organisation that conducts research on polymers states that the number of chemicals used in the manufacturing of plastics is only a fraction of those reported by the PlastChem report (https://www.norner.no/news-and-press-releases/second-critical-response-to-the-plastchem-report/). They state that chemicals in food can migrate into plastic and, therefore, are detected in migration and extraction studies. They also indicate that food packaging materials in several regions in the world are regulated separately. For instance, the EU regulation EU 10/2011 on food contact plastics regulates 1046 chemicals.

During the ongoing negotiations for a global plastic treaty, it has been proposed that the Globally Harmonized System (GHS) of Classification and Labelling of Chemicals (CLP) will be used as hazard-based criteria. Key categories under consideration include Carcinogenic, Mutagenic, Reprotoxic (CMR) substances, chemicals causing specific organ toxicity with chronic toxicity after single or repeated exposure, endocrine disrupting chemicals, as well as PBTs or very persistent and very bioaccumulative (vPvBs). Additional criteria like neurotoxicity, immunotoxicity, and mobility may be introduced in the future.

One of the overarching goals of the Partnership for the Assessment of Risks from Chemicals (PARC) activities is to address the regulatory needs for reliable chemical risk assessment. This is achieved with the active involvement of the European Food Safety Authority (EFSA), the European Chemicals Agency (ECHA), and the European Environment Agency (EEA), and national authorities in reviewing project proposals and providing feedback on deliverables. The Norwegian government, supported by the Norwegian Environment Agency as competent authority for Registration, Evaluation, Authorization and Restriction of Chemicals (REACH), has during the last years supported several initiatives to address the topic of chemicals in plastics within the ongoing United Nations (UN) plastic treaty negotiations, including the screening of the most hazardous chemicals in plastics, as proposed in this project. The PlasticLeach project started in May 2025 and will end in April 2029, bringing together 16 partners across Europe and Korea. PARC is a co-funded project from the European Commission, aimed at improving chemical risk assessment in Europe by advancing biomonitoring, hazard identification and characterization and the science-to-policy translation in an integrated manner. Within the hazard identification and characterization activities, the focus is on the development of New Approach Methodologies (NAMs) for endpoints such as developmental neurotoxicity, immunotoxicity and endocrine disruption. Filling data gaps on the hazards of chemicals is also a key objective of PARC, and the PlasticLeach project directly contributes to this goal.

**FIGURE 1:**
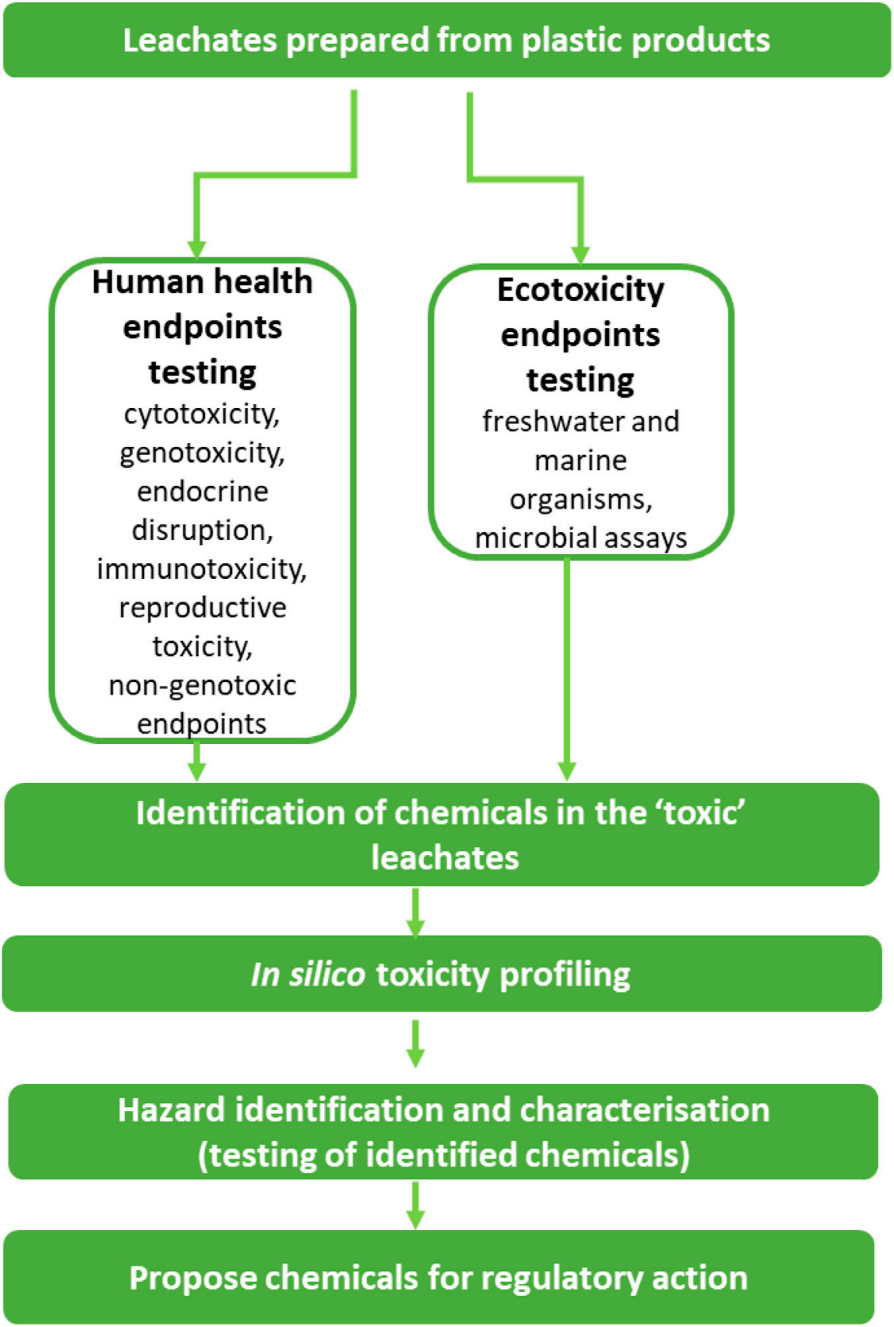
PlasticLeach workflow for identifying, screening hazardous chemicals in plastic products.

The primary objective of the PlasticLeach project is to screen the most hazardous chemicals and their mixtures present and released by different types of plastics. This will be achieved through a tiered approach that includes: (i) testing plastic leachates in biological test systems, (ii) identification of the chemicals present in the leachates, and (iii) conducting further hazard identification and characterization of the identified chemicals using additional testing of individual chemicals after *in silico* profiling. The overall strategy to be applied in PlasticLeach will be mainly based on the use of NAMs, contributing to the European strategy to reduce animal testing for chemicals. This is summarized in Figure 1 and will be described in detail in this paper.

## 2 Selection of plastic products for testing

In the PlasticLeach project, we aim to screen the hazardous chemicals present in a range of products commonly used in everyday life. The selection of these products was based on two main criteria: polymer type and the use category.

The selected polymer types include silicone, polypropylene (PP), polyethylene (PE), polyamide (Nylon), polyethylene terephthalate (PET), polyvinyl chloride (PVC), polylactic acid (PLA) and cellulose polymers. Products containing these polymers are kitchen products, toys, personal care products, clothing, tubes, food contact materials, and medical devices. Special attention was given to products intended for infants and young children, as these groups are considered particularly vulnerable to chemicals due to their developing physiology and, in some cases, higher exposure.

Since the list of plastic items to test was initially too ambitious, we have reduced the initial list to 6 polymers, i.e., fossil fuel-based plastics with long aliphatic groups, fossil fuel-based plastics with more complicated side groups, biobased plastic, biodegradable plastic, silicon and rubber. One consumer product containing each of the polymers will be bought centrally and one laboratory in the consortium will be responsible for preparing the extracts and the distribution of the leachates to the laboratories involved in the testing. Table 1 details the selected polymers and the consumer product that were selected.

**TABLE 1:** Selected polymers and selected consumer products that will be tested in PlasticLeach.

Class	Polymer	Product
Fossil fuel plastic based with long aliphatic groups	Polypropylene	Baby bottles
Fossil based plastic with more complicated side groups	Polyamide	Athletic clothing
Biobased plastic	Polylactic acid	Children cuttery
Biodegradable plastic	Cellulose	Cigarette filters
Silicone	Silicone	Cupcake forms
Rubber	Rubber	Rubber granulates

Rubber granulates derived from recycled car tires will be included in all assays. These granulates are commonly used as infill material in artificial football fields but will be restricted in the EU from 2031 due to their identification as a significant source of micro- and nanoplastic (MNP) pollution in the environment. Since tire wear is an important source of airborne and surface water contamination, exposure of humans and wildlife to rubber particles and associated chemicals is considered likely (Foscari et al., 2025; Wagner et al., 2018). Rubber is a mixture of synthetic and natural polymers, and preliminary ecotoxicological investigations have identified a number of chemicals that can leach from rubber, including the antioxidant N-(1,3-dimethylbutyl)-N′-phenyl-p-phenylenediamine (6PPD), whose breakdown product (6-PPD-quinone) was found to be toxic for certain salmon species (Lo et al., 2023). However, relatively little is known about the effects of chemicals present in rubber on human-relevant test systems.

## 3 Preparation of leachates

In literature, several methods to prepare leachates are reported. Some approaches use strong organic solvents like hexane or acetonitrile to maximize the extraction of chemicals from the plastics, while others use aqueous media or fluids simulating physiological conditions (e.g., digestive fluids like sputum, gastric fluid or artificial saliva) to mimic realistic exposure scenarios (Li et al., 2022; Omidoyin and Jho, 2024; Peters et al., 2022; Lee et al., 2025). For containers and packaging intended for foods, their elution can be utilized with standard food simulants, in accordance with the established procedures relating to food safety (EUR, 2025).

To minimise the co-extraction of non-intentionally added substances such as non-polymerised plastic monomers or intermediates from the polymerisation, PlasticLeach will use a mild organic solvent like methanol to prepare the leachates. Methanol has been used with success for the extraction of intentionally added chemicals such as endocrine disrupting chemicals from plastic food packaging materials (Stevens et al., 2024). In this context, an extraction protocol and a standard operating procedure will be established. Briefly, plastic products will be cut into small pieces to facilitate extraction, using clean stainless-steel blades or a plastic-cutting saw to prevent cross-contamination, under controlled laboratory conditions. Subsequently, internal standards (ISs) will be added to counteract any potential experimental variability and enable quantitative analysis. ISs will be selected after a preliminary chemical screening (see section 5) and will constitute deuterated compounds representative of the most frequently detected chemical groups. Samples will be shaken for 24h in the dark to avoid any potential photodegradation. After extraction, the leachates will be filtered and concentrated under a nitrogen stream, followed by reconstitution and filtering. For biological testing, the majority of leachates will be reconstituted in dimethyl sulfoxide (DMSO) to ensure compatibility with *in vitro* assays. Since contamination from laboratory equipment and disposables is likely to occur, appropriate blanks will be collected during the preparation of the leachates and analyzed alongside the leachates to control background contamination. Pending on the results obtained additional solvents will be considered to prepare leachates, like a non-polar solvent.

## 4 Test battery

To comprehensively assess the potential hazards associated with chemicals leaching from plastic products, a tiered testing strategy will be implemented. The test battery combines *in vitro* assays that cover a broad spectrum of toxicological endpoints, including both general and mechanistic specific effects. This approach ensures the identification of cytotoxic, genotoxic, and non-genotoxic hazards, as well as endocrine-disruptive, reproductive and immunotoxic properties.

Endpoints of both human and environmental relevance are within the scope of PlasticLeach, and project partners have proposed a broad and diverse set of assays appropriate to identify several different mechanisms of toxicity and regulatory-relevant endpoints. For logistical reasons these tests will be conducted according to a tiered approach.

Tier 1 will focus on broad screening to identify generic indicators of potential hazardous properties and prioritize substances for further investigation using mammalian and ecotoxicological assays. It includes assays screening for general cytotoxicity, as well as key regulatory endpoints such as carcinogenicity, mutagenicity, and reproductive toxicity (CMR), in addition to endocrine disruption.

Specifically, cell viability will be evaluated using the Cell Painting assay and MTT assays using human cells. Cell viability will also be evaluated in the rainbow trout gill cell line RTgill-W1 and the Atlantic salmon gill cell line ASG-10 using a combination of three viability assays, Alamar Blue, CFDA-AM and Neutral Red which cover a range of endpoints and are the basis of the OECD Test nr. 249 to predict fish acute toxicity. Genotoxic potential will be screened by the Ames test (according to TG 471) and the *in vitro* micronucleus assay (OECD TG 487). Reactive oxygen species (ROS) formation will be measured in HepG2 cells, and the gill cell lines RTgill-W1 and ASG-10 using 2′,7′-dichlorodihydrofluorescein-diacetate staining. Endocrine activity will be assessed through receptor assays for estrogen, androgen, glucocorticoid, thyroid hormone (THRα and THRβ), and farnesoid X receptors. These tests will be performed according to two OECD test guidelines on androgen and estrogen receptor activities (OECD TG 458 and 455 respectively). Additional endocrine endpoints will be tested using the H295R steroidogenesis assay, in line with OECD test guideline TG 456. The yeast estrogen screen (YES) assay will be employed to screen potential endocrine-disrupting chemicals in leachates. This assay uses a genetically modified *Saccharomyces cerevisae* yeast strain with a human estrogen receptor. In case the yeast is exposed to plastic leachates containing compounds with estrogenic activity, the β-galactosidase enzyme will be expressed and catalyze the degradation of a chromogenic substrate, resulting in a color change.

The (DR) CALUX assay uses rat (H4IIE) hepatoma cells transfected with a luciferase reporter gene to detect dioxins and dioxin-like compounds, such as polychlorinated dibenzofurans (PCDFs) and certain polychlorinated and polybrominated biphenyls (PCBs and PBBs, respectively). These compounds activate the aryl hydrocarbon receptor (AhR), which binds to dioxin-responsive elements, (Murk, 1996).

Ecotoxicological effects will be assessed using the acute immobilization test with *D. magna* and the Microtox test with the bioluminescent bacteria *A. fischeri*. In the acute test, the number of immobilized *Daphnia magna* neonates will be recorded at 24h and 48h of exposure, while in the Microtox test, the light emission by *Aliivibrio fischeri* is measured. Furthermore, the inhibition of the growth of *Caenorhabditis elegans* will be evaluated by measuring body length at 48 h.

A detailed list of proposed assays included in Tier 1 is provided in Table 2.

**TABLE 2:** Tier 1 assays that will be performed on leachates prepared from plastic products. The partner responsible for conducting the assay is indicated in brackets.

Endpoint	Test	References
Cell viability	Cell- painting (NIPH) (under development) MTT tests in HepG2 cell line (BPI) Fish gill cell lines (Alamar Blue, CFDA-AM and Neutral Red) (NVI)	Smith et al. (1996) Solhaug et al. (2024) OECD TG 249
Genotoxicity	Ames test MPF (IEP-NRI) UmuC (IEP-NRI) *In vitro* micronucleus (INSA)	OECD TG 471 ISO 13829 OECD TG 487 (2023) Vasconcelos et al. (2019)
Nuclear receptor reporter assays	DR CALUX assay (WU) THRα, THRβ, FXR (KIST) Androgen receptor (INRS) Estrogen receptor (INRS) Glucocorticoid receptor (ULFFA) Yeast Estrogen Screen (YES) assay (UAVR)	Murk (1996) Choi et al. (2025) OECD TG 458 OECD TG 455 (Kenda et al., 2020) Gomes et al. (2023)
Reactive Oxygen Species (ROS) formation	HepG2 and fish gill cell lines (RTgill-W1, ASG-10) cells with 2′,7′-dichlorodihydrofluorescein-diacetate staining (NVI) BPI	Solhaug et al. (2023)
Steroidogenesis	H295R steroidogenesis assay - (KIST) INRS	OECD TG456
Ecotoxicity	Acute immobilisation test *Daphnia magna* (KIST, IEP-NRI) Microtox test (UAVR, IEP-NRI) *Raphidocelis subcapitata* growth inhibition tests (IEP-NRI) *Caenorhabditis elegans* inhibition growth assay (body length) (UPO)	OECD TG 202 Cardoso et al. (2023) OECD TG 201 Lucanic et al. (2018)

In Tier 2 of the testing strategy, supplementary tests will be performed to provide a more detailed evaluation of specific toxicological endpoints not fully covered in Tier 1. These additional assays offer deeper insights into the genotoxic, immunotoxic, endocrine, and ecotoxic potential of chemicals leaching from plastics. For example, genotoxicity will be assessed using assays such as the *in vitro* comet test, γ-H2AX assay, mammalian Hprt gene mutation assay (OECD TG 476), and the UmuC test. Immunotoxic effects will be assessed using a THP-1 immune cell model to investigate the trigger of innate immune response and the adaptive immune system using Jurkat T cell and lympholastoid cell lines (LCL) as *in vitro* models. If plastic leachates affect the intestinal barrier and immune responses will be studied using a co-culture model of THP-1 derived macrophages and Caco-2 intestinal epithelial cells. This model allows to study the crosstalk between epithelial and immune cells, providing insights into inflammation and barrier integrity disruption.

Disruption of the intestinal barrier and effects on immune-metabolic health will be determined with an advanced co-culture model using THP-1 cells, CaCo2 cells and hMSC-derived adipocytes. Obesity induction and metabolic effects will be investigated in *C. elegans*. Transcriptomics assessment in *Caenorhabditis elegans* will be carried out with transgenic strains expressing GFP under the control of specific reporter genes for obesity and metabolic changes. Synchronized 1 day old larvae are dispensed into 96-well plates and exposed to the leachates in M9 medium containing *E. coli* OP50 bacteria at 20 °C for 72 h. Sodium azide is added to immobilize the larvae for fluorescent micrograph imaging and quantification (Papsdorf et al., 2023). Additional endpoints include cell viability and proliferation in advanced 3D liver models, biocompatibility testing with mouse fibroblasts following ISO standards, and transformation potential via the Bhas 42 cell transformation assay.

Table 3 provides a detailed overview of the Tier 2 assays.

**TABLE 3:** Tier 2 assays that will be performed on leachates prepared from plastic products. The partner responsible for conducting the assay is indicated in brackets.

Endpoint	Test	References
Genotoxicity	Comet test *in vitro* (WU) Gamma H2AX assay (BPI) Mammalian Hprt gene mutation assay (INSA) UmuC (IEP-NRI)	Olive and Banáth (2006) Khoury et al. (2013) OECD TG 476 (2016) (Vital et al., 2025) ISO 13829
Immunotoxicity	Immunetoxicity and cell barrier function disruption (UU) Intestine (health and inflammation) mono/co-culture model using THP-1 monocytes, and Caco2/HR29 cells (WUR) Jurkat T cells (ULFFA)	Paul et al. (2023), Lehner et al. (2020) Brouwer et al. (2024) Busch et al. (2021) Franko et al. (2025)
Metabolic	Transcriptomics assessment of obesity and metabolic effects in *Caenorhabditis elegans* with reporter genes (UPO)	Papsdorf et al. (2023)
Viability, proliferation, genotoxicity	3D HepG2 model (NIB) Cytotoxicity: ATP assay (NIB) Genotoxicity: Gamma H2AX assay (BPI), pH3 assay (NIB)	Štampar et al. (2022) Sendra et al. (2023) Sendra et al. (2023)
Biocompatibility	Mouse fibroblasts (NIB)	ISO 10993-5 (2009)
Cell Transformation assay	Bhas 42 cell transformation assay (CTA) (INRS)	Fontana et al. (2017)

In Tier 3, follow-up testing of selected Tier 1 and Tier 2 will be performed, along with additional tests that require larger quantities of test materials than initially produced.

Functional alterations induced by the plastic leachates in bovine, human and sea urchin sperm cells will be assessed by evaluating the sperm viability and motility parameters. Sperm viability will be determined by using colorimetric methods which will enable the measurement of viable cells. Sperm motility will be evaluated using the Sperm Class Analyzer® CASA System (Microptic) equipped with SCA v5.4 software, a computer-assisted platform.

Potential effects of plastic leachates on fertilization success and early developmental stages of the sea urchin *Paracentrotus lividus* will be assessed. Eggs and sperm from sea urchins will be collected and used for *in vitro* fertilization. Fertilized embryos will be exposed to plastic leachates, and by the end of the incubation period, the embryonic development will be assessed for morphological abnormalities.

Potential disruption of the thyroid hormone system can be assessed by quantifying thyroxine (T4) levels in the zebrafish embryo model. Zebrafish embryos will be exposed up to 120 h post-fertilization (hpf). During this developmental period, zebrafish are not yet capable of independent feeding and are therefore considered an *in vitro* model for testing purposes (Bauer et al., 2021). In this system, T4 concentrations can be determined in whole-body homogenates (Jaka et al., 2023; Jia et al., 2016), intrafollicular T4 can be visualized in whole-mounts (Rehberger et al., 2018) and gene expression changes of key thyroid-related genes can be evaluated to provide mechanistic insights (Gölz et al., 2025).

Disruption of organismal development and physiology due to plastic leachates will be assessed by examining life history traits of *Caenorhabditis elegans*. *C. elegans* is a well-established invertebrate model organism with a short life cycle and transparent body, allowing detailed observation of developmental and reproductive endpoints. Synchronized populations of *C. elegans* will be exposed to defined concentrations of plastic leachates from the L1 larval stage onward (for 24h). At the close of the exposure period, life history traits such as growth rate, fertility, fecundity, and lifespan will be quantified as sensitive indicators of toxic stress. In this system, phenotypic measurements can be complemented by molecular analyses (e.g., stress-response and reproduction-related gene expression/protein content) to elucidate potential mechanisms underlying the observed effects.

Neurotoxic effects in *C. elegans* will be evaluated by measuring locomotive activity with an array of micro beams of infrared light. L4-stage larvae are exposed in M9 buffer into each well in a 96-well flat microtiter plate. All recordings are analyzed in 30-min time bins (Hahnel et al., 2021) Oxidative stress and other transcriptomic changes will be evaluated as described in tier 2.

An overview of Tier 3 assays can be found in Table 4.

**TABLE 4:** Tier 3 assays that will be performed on leachates prepared from plastic products. The partner responsible for conducting the assay is indicated in brackets.

Endpoint	Test	References
Endocrine	Intrafollicular thyroxine (T4) using zebrafish embryo (BPI)	Raldúa and Babin (2009) Rehberger et al. (2018)
Developmental and Reproductive Toxicology	Sperm cells viability and motility assays (UAVR) *Paracentrotus lividus* embryo development test (UAVR) *Daphnia magna* reproduction test (IEP-NRI) *Caenorhabditis elegans* life history traits (lethality, fecundity, fertility and others) (BPI)	Silva et al. (2021) Ravera et al. (2006) OECD TG 211 Kuo et al. (2025)
Neurotoxicity	Neurotoxicity, oxidative stress and other effects in *Caenorhabditis elegans* (UPO)	Hahnel et al. (2021)

## 5 Identification of chemicals in leachates by non-target screening (NTS)

Leachates that were tested and caused hazardous effects will be examined by an exploratory method aiming to detect and identify unknown chemicals without prior assumptions. The workflow includes:

- Chemical analyses with liquid chromatography coupled to high-resolution mass spectrometry or GCMS.
- Peak detection: Identifying unknown peaks in high-resolution mass spectra.
- Molecular formula generation: Using isotope patterns and exact mass;
- Spectral deconvolution and database searching: Comparing against public/private spectral libraries, like for example, the NORMAN database;
- Structure elucidation: Using fragmentation data and computational tools (e.g., *in silico* prediction) as well as comparison with commercially available standards of the candidate substances.

It is anticipated that the proposed workflow will help to identify novel contaminants, transformation products and unexpected chemicals. The approach can be time-consuming, with complex data interpretation and potential for false positives. In the mass spectrometric measurements of plastic extracts, commonly about 500 features can be observed but only 1 or 2% are identified with high certainty. The need for harmonized processes for data processing and chemical identification, as well as the development of a standardised workflow, was identified. This will be discussed with other partners involved in NTS activities in other projects in PARC.

Also in this respect, procedural blanks are needed to avoid false positives, and replicates of leachates will be used to consolidate findings.

## 6 In silico-approaches

The leachates are a mixture of chemicals and will be tested in the Tier 1-3 assays. Chemicals present in the most hazardous mixtures will be identified as described under 5. Identified individual chemicals or chemicals that are suspected to be present in the leachates will be screened for hazard identification by *in silico* approaches using the most up to date databases and tools. Databases will include TOXCAST for human-focused mechanistic data, and ECOTOX knowledgebase for environmental effects data, supplemented by QSAR effects predictions from the OECD QSAR toolbox. If available, artificial intelligence based tools for hazard identification, as, for example, being developed in the ONTOX project, will be employed as well (Kleinstreuer and Hartung, 2024). *In silico* hazard identification shall facilitate adjusting the individual chemicals and prioritization of assays for additional testing of individual chemicals and the interpretation of experimental results. Moreover, the heterogeneous set of hazard-related data will be summarized, e.g., by deriving scores for each type of hazard endpoint like endocrine, genotoxicity and reproductive toxicity, combining data from both human and ecotoxicological assays where natural. In cases where integration of such data is not feasible, separate hazard scores will be developed. An overall score for concern can then be derived by summing scores from individual types of hazard data as well as other relevant data such as production volumes as proximate for exposure, as available from PlastChem DB (https://plastchem-project.org/).

Generic PBK modeling can be used for quantitative *in vitro* to *in vivo* extrapolation (QIVIVE). These models apply a standardized compartmental structure with species-specific physiological parameters (e.g., organ volume, blood flow rates), while chemical-specific parameters are derived from *in vitro* human or wildlife biokinetic assays (e.g., metabolic rates) and predictive *in silico* tools (e.g., tissue partition coefficients) such as QSAR (“Guidance document on the characterisation, validation and reporting of Physiologically Based Kinetic (PBK) models for regulatory purposes,” OECD, 2021) (Fragki et al., 2022; Grech et al., 2019). This approach allows for estimation of human and wildlife dose-response relationships by relating external doses to clinically or population-relevant adverse effects, based on *in vitro* concentration-effect relationships at the cellular level. Generic PBK models provide a useful tool for simulating toxicokinetics, however challenges remain in predicting absorption and clearance of highly lipophilic as well as ionized compounds, as well as the kinetics of chemical mixtures, which need to be addressed.

## 7 FAIR data principles

**FIGURE 2:**
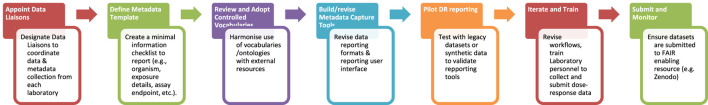
Implementation of FAIR principles within the PlasticLeach project.

The FAIR (Findable, Interoperable, Accessible and Reusable) principles are a central element in all experimental work within PARC to ensure standardized procedures for the storage of metadata and observational data for use by regulatory agencies and reuse by other scientists. These principles facilitate regulatory review and enable the wider scientific community to access and apply the data. In the PlasticLeach project, a dedicated data reporting user interface (UI) for quantitative dose (concentration)-response data, qData (https://doi.org/10.5281/zenodo.16942844) has been developed to facilitate reporting metadata and observational data from *in vitro* and *in vivo* studies and act as a FAIRification hub for further reuse of data. The key steps of implementing FAIR principles in PlasticLeach are depicted in Figure 2.

## 8 Integration of data from the suite of bioassays

Standardised dose-response data from the *in vitro*, *in vivo* and *in silico* approaches on proposed will be subjected to statistical analysis and dose-response modeling to derive No Observed Effect Concentration (NOEC), Lowest Observed Effect Concentration (LOEC) and benchmark dose (BMD) as points of departure (POD) from normality. These PODs on both mixtures and individual chemicals will be compared across bioassays, species and endpoints to identify the most susceptible targets spanning the human health and ecotoxicological bioassays employed. *In vitro* to *in vivo* extrapolation (IVIVE) will be explored as a means to compare effects across bioassays and provide safe thresholds for the suite of organisms tested. These thresholds for no effect can be used to identify potential toxicity drivers (i.e., chemicals with the largest hazard potential), susceptible species and most relevant mode of action (MoA) for the complex leachate mixtures and individual chemicals quantified in these mixtures. The overall outcome of PlasticLeach will be a list of the most hazardous chemicals present in plastic products that will be presented to regulatory agencies for possible future regulatory action.

## 9 Discussion

The United Nations identified a triple planetary crisis in 2022, consisting of climate change, pollution, and biodiversity loss. These crises are deeply interconnected, and effective mitigation requires knowledge-based actions.

One emerging concern is the predicted increase in the production of plastics. Both the formation of micro- and nanoplastics and the release of chemicals from plastics are of concern to both human health and the environment and contribute to the triple planetary crisis. Although chemicals are integral to plastic production, several sources recognise a significant knowledge gap regarding which chemicals are present in plastics, their potential release and possible hazardous effects. Exposure to these chemicals is widespread as evidenced by European biomonitoring initiatives and data demonstrating their presence in the environment.

Several reports have indicated the urgent need for additional hazard assessment on chemicals present in plastics. However, manufacturers are generally not required to generate such data unless specifically mandated under the REACH regulation in Europe, which is primarily based on production tonnage. Currently, the exact chemical composition of many plastic products remains unknown. The PlasticLeach project aims to address this gap by screening for the presence of hazardous chemicals in plastic products using a tiered testing approach. Initial data will be integrated using criteria from the CLP (Classification, Labelling and Packaging) Regulation, focusing on endpoints such as carcinogenicity, mutagenicity, reproductive toxicity (CMR), immunotoxicity and endocrine activity. It is anticipated that the identified toxicity from the leachates is most likely the result of a mixture of chemicals. To better understand these effects, attempts will be made to identify individual chemicals within the mixtures and test them separately to determine relative potency factors and assess the total risk from the simultaneous exposure to coinciding chemicals. For the assessment of mixture toxicity, the dose (concentration) addition (DA or DC) model or the Independent Action will be considered based on available data on Mode of Action (MoA). Knowledge of the mode-of-action of chemicals, for example, by comparing Molecular Initiating Events from Adverse Outcome Pathways, would allow the reduction of the uncertainty in the estimation of the risk from the combined exposure to chemicals.

If hazardous chemicals or mixtures are identified, regulatory action might be needed. The Norwegian government has indicated its willingness to take a leading role in this process when appropriate.

Every project has its own risk profile, and some of the identified risks are:

- Insufficient capacity within the consortium to produce enough leachates for comprehensive biological testing
- Potential contamination with chemicals from laboratory equipment, as much of this equipment is made from plastics. While blanks will be included to control for this contamination, this will increase the total number of samples requiring testing and analysis.
- Challenges in identifying unknown chemicals within complex mixtures, can be both technically demanding, time-consuming and costly.

The data generated by this project will be used to support the ongoing discussions on a UN plastic treaty. In an ambitious plastic treaty, we need to have more knowledge on the chemical composition of plastics, reduce the volume of plastic produced and increase recycling. In addition, the findings can inform the Science-Policy panel on the sound management of chemicals and waste, as well as pollution prevention initiatives.

## Data Availability

The original contributions presented in the study are included in the article/supplementary material, further inquiries can be directed to the corresponding author/s.
